# Enhanced vs. standard low dose dexamethasone treatment on respiratory outcomes of preterm infants with bronchopulmonary dysplasia

**DOI:** 10.3389/fped.2025.1603308

**Published:** 2025-08-07

**Authors:** Sezgin Gunes, Suzan Sahin, Ozlem Bozkurt, Begum Cezayir, Arda Bozgul, Deniz Gonulal, Mehmet Yekta Oncel

**Affiliations:** ^1^ Department of Neonatology, Buca Seyfi Demirsoy Teaching and Research Hospital, Izmir, Türkiye; ^2^ Faculty of Medicine, Department of Pediatrics, Division of Neonatology, Izmir Democracy University, Izmir, Türkiye; ^3^ Faculty of Medicine, Department of Pediatrics, Division of Neonatology, Kocaeli University, Izmit, Türkiye; ^4^ Department of Neonatology, Medical Point Hospital, Izmir, Türkiye; ^5^ Department of Neonatology, Izmir City Hospital, Izmir, Türkiye; ^6^ Faculty of Medicine, Department of Pediatrics, Division of Neonatology, Izmir Katip Celebi University, Izmir, Türkiye

**Keywords:** premature infants, bronchopulmonary dysplasia, systemic corticosteroids, neonate, dexamethasone

## Abstract

**Background:**

Premature infants are particularly vulnerable to complications such as bronchopulmonary dysplasia (BPD). Although systemic steroids have been used for some time, no effective alternative treatments have emerged. This has led clinicians to investigate various dosing regimens and discuss the necessity of revising systemic steroid dosages. This study aims to evaluate the effectiveness and safety of two different doses of systemic dexamethasone use in treating BPD in premature infants born at or below 30 weeks of gestation.

**Methods:**

A retrospective review was conducted on hospital records of premature infants admitted to the Neonatal Intensive Care Unit of Izmir Private Medicalpark Hospital for a 6-year period. In the first 2 years of these 6 years, as historical controls, infants with evolving BPD had received dexamethasone treatment according to the DART protocol, while the ones born in the last 4 years received enhanced low-dose treatment. Data collected included demographic characteristics, risk factors for preterm birth, duration and way of oxygen support, prematurity-related complications, systemic dexamethasone doses, and mortality.

**Results:**

Of the 368 infants analyzed in the study, 265 had received systemic enhanced low dose dexamethasone, while 103 had received low dose dexamethasone treatment. Baseline characteristics including gestational age, birth weights, and RDS severity were similar among groups The study found that a cumulative dose of 1.35 mg/kg of systemic dexamethasone (enhanced low dose) is both safe and more effective than standard low dose dexamethasone (DART protocole) therapy in the management of BPD. The DART group required significantly longer invasive and non-invasive ventilatory support compared to the enhanced low-dose group. By the 36th gestational week, a higher proportion of infants in the enhanced low-dose group were free from oxygen support. The enhanced low-dose group also had a higher extubation success rate. Dexamethasone -related side effects were minimal, with only two cases of hypertension and one of interventricular septal hypertrophy in the enhanced low dose group, and one case of hypertension in the DART group, all resolving over time.

**Conclusions:**

Our study suggests that an enhanced low-dose dexamethasone regimen (1.35 mg/kg) may offer superior respiratory outcomes compared to the conventional DART protocol without a significant increase in short-term adverse effects.

## Background

Bronchopulmonary dysplasia (BPD) remains a leading cause of morbidity among premature infants. Advancements in survival rates among extremely premature infants have led to an increased incidence of BPD and prolonged mechanical ventilation (1). Although its etiopathogenesis is not fully elucidated, it is recognized as multifactorial, with primary contributing factors including barotrauma and/or volutrauma associated with mechanical ventilation, as well as inflammation. Recent studies also underscore the role of perinatal factors in initiating the inflammatory cascade prior to birth (2–4). In an effort to mitigate this inflammatory process, both systemic and inhaled corticosteroids have been widely employed (5–10).

Corticosteroids are among the most extensively investigated pharmacologic agents in neonatology, with over 80 randomized controlled trials conducted to date. Nevertheless, fundamental questions persist regarding the optimal agent, timing of initiation, dosage, and duration of therapy (11). While systemic hydrocortisone has demonstrated efficacy in reducing mortality and necrotizing enterocolitis (NEC), its impact on the incidence of BPD appears limited (12). In contrast, dexamethasone is more commonly utilized worldwide and is classified according to timing of initiation (early: 0–7 days; moderately early: 8–14 days; late: 15–27 days) and cumulative dose (low: <2 mg/kg; medium: 2–4 mg/kg; high: >4 mg/kg) (13).

The Dexamethasone: A Randomized Trial (DART) protocol advocates for a low-dose regimen with a cumulative dose of 0.89 mg/kg over 10 days (14). However, as the DART trial was prematurely discontinued after enrolling only 70 participants, its statistical power to detect significant differences in outcomes is limited. The Turkish Neonatology Society (TNS) currently advises against early systemic corticosteroid administration (<7 days of life) but supports late systemic dexamethasone therapy for infants at risk of developing severe BPD, provided that they are ventilator-dependent with an FiO₂ requirement exceeding 40% for at least two weeks and that comorbidities such as late-onset sepsis, patent ductus arteriosus (PDA), or intraventricular hemorrhage (IVH) have been excluded. The TNS endorses the DART protocol as the standard treatment approach in such cases (15).

Systemic dexamethasone administered after the first week of life has been shown to reduce the incidence of BPD, mortality, and the need for PDA treatment; however, it may also lead to adverse effects such as hyperglycemia, hypertension, and hypertrophic cardiomyopathy (16, 17). Given the ongoing uncertainty regarding the optimal dose, timing, and duration of therapy, and the limitations of existing studies (13, 18, 19), individualized and judicious use is essential to maximize benefit while minimizing harm. A recent systematic review and network meta-analysis of 62 studies concluded that moderately early initiation of systemic dexamethasone at a medium cumulative dose (2–4 mg/kg) was associated with the greatest reduction in the composite outcome of mortality or BPD, although the overall quality of evidence was low (3, 20).

Recent comparative studies have begun to investigate variations within low-dose regimens. Al-Taweel et al. compared the standard DART protocol with an enhanced low-dose regimen (approximately 1.3 mg/kg) and reported improved respiratory outcomes without a significant increase in adverse events, suggesting that even within the low-dose range, dosing adjustments may yield clinically meaningful differences (21). Similarly, a meta-analysis by Onland et al. had found that higher cumulative doses of dexamethasone were more effective in reducing pulmonary morbidity, but the risk of neurodevelopmental impairment increased with higher exposure, underscoring the need for cautious optimization of dosing strategies (22).

In clinical practice, steroid regimens for BPD vary significantly across centers, with no universally accepted standard. The cumulative dose recommended by the DART protocol is frequently perceived as insufficient in achieving meaningful clinical improvement. Based on our institutional experience, the standard low dose of 0.89 mg/kg often fails to produce the desired therapeutic response in infants diagnosed with BPD.

This retrospective study aims to compare the outcomes of premature infants treated with a cumulative low dose of 1.35 mg/kg dexamethasone to historical data from those treated according to the DART protocol. By evaluating both respiratory outcomes and prematurity-related complications, we seek to determine whether a modest increase in the low-dose regimen may offer enhanced clinical efficacy without incurring additional risk, thereby contributing valuable data to the evolving body of literature on systemic corticosteroid use in BPD management.

## Materials and methods

The hospital records of all premature infants who born at or below 30 weeks of gestation, and were admitted to the Neonatal Intensive Care Unit (NICU) of Izmir Private Medicalpark Hospital during the 6-year period were retrospectively reviewed. In this clinic, during the last four years of the studied period, systemic steroid therapy was routinely administered to eligible infants at twice the dose specified in the DART protocol, which still fell within the low-dose regimen range (enhanced low dose). Before these four years, however, when steroid treatment was required for the similar group of infants due to evolving BPD, the treatment dose was planned in accordance with the DART protocol (low dose). In order to compare the results of two different dosing regimens, historical controls were recruited to the study. For historical controls, a two-year retrospective analysis was conducted. Data on the demographic characteristics of the infants and their mothers, risk factors for preterm birth, duration of oxygen support and other respiratory outcomes, prematurity-related complications [such as NEC, retinopathy of prematurity (ROP), IVH, etc.], the systemic steroid dose and number of courses administered for BPD treatment, as well as mortality outcomes, were recorded.

Ethics approval was obtained from the Buca Seyfi Demirsoy Teaching and Research Hospital Ethics Committee. As a retrospective study, all data were anonymized and used strictly for research purposes.

The inclusion criteria for the study were being born at or below 30 weeks of gestation and being hospitalized in the mentioned hospital until discharge.

The exclusion criteria for the study included significant deficiencies in medical records, being prescribed a different dose and application route of steroids, having major congenital anomalies, genetic anomalies, being transferred to another hospital for continuation of treatment or death before 36th gestational week.

A total of 667 preterm infants with gestational ages between 23 and 30 weeks were evaluated. Due to the exclusion criteria, 299 infants were excluded, predominantly due to missing data in their medical records, including 15 who had received a corticosteroid regimen with a different dose and/or route. All the data investigated were extracted from medical records.

In our study, consistent with our routine approach, the definition and severity classification of BPD were made according to the classification proposed by Jobe AH and Bancalari E at the 2001 National Institutes of Health (NIH) Workshop, which remains the most widely used definition today. BPD is classified based on gestational age at birth and the degree of respiratory support required at specific postnatal time points. For infants born at less than 32 weeks' gestation, the evaluation occurs at 36 weeks postmenstrual age (PMA) or at discharge, whichever comes first. In this group, mild BPD is defined as an oxygen requirement of ≥21% for at least 28 days but no supplemental oxygen need at the time of assessment. Moderate BPD involves a need for <30% supplemental oxygen, while severe BPD includes a requirement of ≥30% oxygen and/or the need for positive pressure support such as nasal continuous positive airway pressure (nCPAP) or positive pressure ventilation (PPV). For infants born at or beyond 32 weeks' gestation, evaluation is performed between postnatal days 28 and 56 or at discharge. The criteria for mild, moderate, and severe BPD mirror those used for infants <32 weeks, adjusted for the older evaluation age. This classification system enables standardized assessment of BPD severity across different gestational ages (2).

As a routine practice in the unit, systemic steroid treatment was initiated for all infants if they were ventilator-dependent with >40% FiO2 for at least two weeks. Infants requiring any kind of respiratory support (even free-flow oxygen) on the 28th day of life also received steroid treatment. According to the unit protocol, only intravenous dexamethasone is employed for BPD. The use of other systemic corticosteroids, such as hydrocortisone, was limited to cases of vasopressor need due to hypotension.

In the enhanced low dose steroid group, the routine cumulative dose administered to each preterm infant with BPD was 1.35 mg/kg of intravenous dexamethasone, which is higher than the dose of the DART protocol (0.89 mg/kg) but still falls within the low cumulative dose category. This cumulative dose was calculated for each infant, and those who received a different dose were excluded from the study. Per the unit protocol, the routine intravenous dosages applied are as follows: on days 1, 2 and 3: 0.2 mg/kg/day; on days 4, 5, and 6: 0.15 mg/kg/day; and on days 7, 8, and 9: 0.1 mg/kg/day. During the study period, no additional modifications were made to the routine clinical practices at the unit level, other than the implementation of the enhanced dose regimen. All other aspects of patient management, including monitoring protocols, supportive care measures, and treatment guidelines, remained consistent throughout the study duration.

Feeding intolerance was defined as the occurrence of more than two episodes of gastric residuals exceeding 50% of the previous feeding volume, or the presence of bilious residuals, vomiting, hematochezia, or abdominal distension. In this study, our primary aim was to to compare the respiratory outcomes (such as the day of extubation and oxygen need at 36.weeks, etc). Our secondary aims were to evaluate prematurity-related complications, potential maternal risk factors and the possible side effects of systemic dexamethasone therapy administered higher than the dose of the classic DART protocol.

## Statistical analysis

Statistical analysis was conducted using the SPSS software for Windows version 20.0 (IBM, Chicago, IL, USA). Descriptive statistics were used including mean [with standard deviations (SD)] and median [interquartile range (IQR)] for continuous variables, and counts (proportions) for categorical variables. Student *t*-test and Mann–Whitney *U* test compared continuous variables for parametric and non-parametric variables, respectively. v^2^ or Fischer's exact test was used to analyze categorical variables. Statistical significance was considered if *p* value was <0.05.

## Results

Of the 368 infants analyzed in the study, 265 had received systemic enhanced low dose dexamethasone, while 103 had received low dose dexamethasone treatment due to a diagnosis of BPD. All of the basic demographic characteristics analyzed were similar in between the two groups. The mean gestational age and birth weight (928.6 ± 203.6 g in the DART group and 990.6 ± 301.3 g in the enhanced low dose group) were similar in both groups (*p* = 0.149; *p* = 0.125). The weight at discharge was significantly higher in the DART group (2,747.8 ± 877.4 vs. 2,065.8 ± 971.2 g, *p* = 0.037). Gender distributions were balanced between groups (male: 52% vs. 52%; *p* = 0.978) (Table 1).

**Table 1:** Demographic and clinical characteristics, according to steroid dose received.

Characteristics	DART protocol group (*n*:103)	Enhanced low dose group (*n*:265)	*p*
Maternal age, years	29.8 ± 4.8	29.3 ± 5.3	0.785
Gestational age, week	26.7 ± 1.6	27.2 ± 2.1	0.149
Cesarean delivery, *n* (%)	82 (80%)	201 (76%)	0.657
APGAR 1. min, median	4	5	0.314
APGAR 5.min, median	7	7	0.993
Birth weight, g	928.6 ± 203.6	990.6 ± 301.3	0.125
Weight at discharge, g	2,747.8 ± 877.4	2,065.8 ± 971.2	**0** **.** **037**
Gender of the newborn, male, *n* (%)	53 (52%)	138 (52%)	0.978
Cardiopulmonary resuscitation at birth	17 (16.5%)	38 (14.3%)	0.214
Risk factors for prematurity (*n*)			
Gestational diabetes mellitus	8 (7.8%)	21 (7.9%)	0.994
PPROM	29 (28.1%)	87 (32.8%)	0.114
Preeclampsia	21 (20.4%)	41 (15.5%)	0.217
Chorioamnionitis	4 (3.9%)	7 (2.6%)	0.432
Placental abruption	2 (1.9%)	6 (2.2%)	0.692
Oligohydramniosis	16 (15.5%)	21 (7.9%)	0.067
Polyhydramniosis	0 (0%)	5 (1.9%)	0.419
Smoking	13 (12.6%)	30 (11.3%)	0.367
Multiple pregnancy	27 (26.2%)	83 (31.3%)	0.284
Primigravidity	50 (48.5%)	110 (41.5%)	0.113
Antenatal corticosteroids	83 (80.5%)	202 (76.2%)	0.382

Statistically significant p-values are indicated in bold.

BPD, bronchopulmonary dysplasia; PPROM, preterm premature rupture of membranes.

Maternal risk factors for prematurity, including hypertensive disorders, chorioamnionitis, and premature rupture of membranes, showed no statistically significant differences (all *p* > 0.05). Antenatal corticosteroid administration rates were similar (DART: 80.5% vs. enhanced low-dose: 76.2%; *p* = 0.382) (Table 1).

When the two infant groups are analyzed according to their birth weight categories, 18% of infants in the DART group were small for gestational age (SGA), as this ratio was 12.4% in the enhanced low dose group (*p* = 0.202). Majority of the infants were appropriate for gestational age (AGA) in both groups while IUGR ratios were also statistically similar**.**

The characteristics of dexamethasone therapy timing and administration are summarized in Table 2. The mean day of life at the start of steroid treatment was comparable between groups (23.4 ± 7.1 days in the DART group vs. 22.7 ± 8.0 days in the enhanced low-dose group; *p* = 0.782), as was the mean postmenstrual age at initiation (29.8 ± 3.4 weeks vs. 30.8 ± 4.5 weeks; *p* = 0.554). Per protocol, 32% of infants in the DART group and 35% in the enhanced low-dose group commenced treatment after 28 days of life (*p* = 0.124). Baseline respiratory status was also similar, with invasive ventilation present in 63.1% of the DART group and 64.5% of the enhanced low-dose group at the time of treatment initiation (*p* = 0.892). As expected, the cumulative dose of dexamethasone was significantly higher in the enhanced low-dose group (1.35 mg/kg vs. 0.89 mg/kg; *p* = 0.023), with a similar treatment duration (9.2 ± 1.3 days vs. 10.1 ± 1.1 days; *p* = 0.189) (Table 2).

**Table 2:** Characteristics of dexamethasone treatment timing in infants with evolving BPD.

Parameter	DART protocol group (*n*:103)	Enhanced low dose group (*n*:265)	*p*
Day of life at treatment start, days	23.38 ± 7.13	22.67 ± 8.04	0.782
Postmenstrual age at treatment start, weeks	29.82 ± 3.41	30.81 ± 4.49	0.554
% Started after 28 days (per protocol)	32%	35%	0.124
Baseline respiratory status: invasive ventilation at start	65 (63.1%)	172 (64.5%)	0.892
Cumulative dose received (mg/kg)	0.89	1.35	**0** **.** **023**
Actual duration of treatment, days	10.1 ± 1.1	9.2 ± 1.3	0.189

Statistically significant p-values are indicated in bold.

Prematurity related complications like RDS, feeding intolerance, pneumothorax, sepsis, etc. showed no significant differences between groups (all *p* > 0.05). The infants had received similar number of surfactant repeat doses (*p* = 0,224) (Table 3).

**Table 3:** Prematurity complications and side effects according to steroid dose received.

Complication	DART protocol group (*n*:103)	Enhanced low dose group (*n*:265)	*p*
RDS (moderate or severe)	101 (98%)	258 (97.3%)	0.983
Surfactant doses			
0	2 (1.9%)	7 (2.6%)	0.224
1	35 (33.9%)	72 (27.2%)	
≥2	66 (64%)	186 (70.2%)	
Feeding intolerance	64 (62.1%)	150 (56.6%)	0.332
Pneumothorax	0 (0%)	11 (4.1%)	0.144
ENS	6 (5.8%)	13 (4.9%)	0.890
LNS	62 (60.2%)	145 (54.7%)	0.284
hsPDA	35 (33.9%)	100 (37.7%)	0.866
NEC (stage 2–3)	17 (16.5%)	25 (9.4%)	0.139
IVH (grade 3–4)	9 (8.7%)	22 (8.3%)	0.993
ROP (stage 3–4)	12 (11.6%)	38 (14.3%)	0.301
Steroid induced HT	1 (0.97%)	3 (1.13)	0.778
Steroid induced IVSH	0 (0%)	1 (0.37)	0.923
Steroid induced hyperglycemia	0 (0%)	0 (0%)	1
Steroid induced gastrointestinal bleeding	0 (0%)	0 (0%)	1

RDS, respiratory distress syndrome; ENS, early neonatal sepsis; LNS, late neonatal sepsis; hsPDA, hemodynamicaly significant patent ductus arteriosus; NEC, necrotizing enterocolitis; IVH, intraventricular hemorrhage; ROP, retinopathy of prematurity; HT, hypertension: IVSH, interventricular septal hypertrophy.

Regarding steroid-related side effects, three infants (1.13%) in the enhanced low-dose group developed hypertension, which resolved after treatment cessation, and one infant (0.37%) developed interventricular septal hypertrophy, which also resolved over time (*p* = 0.778; *p* = 0.923). No cases of hyperglycemia, electrolyte abnormalities, or gastrointestinal complications were reported in either group. In the DART group, only one infant (0.97%) experienced hypertension, which resolved spontaneously following steroid withdrawal.

The mean duration of invasive ventilatory support in the DART group was significantly higher than the enhanced low dose group (18.48 ± 14.13 vs. 6.87 ± 13.93 days, *p* = 0.021). Besides this, duration of also noninvasive ventilatory support, total respiratory support, hospitalization, intubation need beyond 28 days were significantly higher in the DART group (*p* = 0.019; 0.033; 0.041; 0.003). On day 28, 32% of the infants in the DART group remained on invasive ventilatory support, while this ratio was 5.7% in the enhanced low dose group. By the end of the enhanced low-dose systemic steroid therapy, significantly higher ratio of infants on invasive ventilatory support before the treatment, were successfully extubated (55%) compared to the ones who received steroid therapy according to the DART protocol (32.5%) (*p* = 0.009) (Table 4).

**Table 4:** Respiratory outcomes of infants.

Outcome	DART protocol group (*n*:103)	Enhanced low dose group (*n*:265)	*p*
Duration of invasive ventilatory support, days	18.48 ± 14.13	6.87 ± 13.93	**0** **.** **021**
Duration of noninvasive ventilatory support, days	20.82 ± 12.31	14.91 ± 14.69	**0** **.** **019**
Duration of total respiratory support, days	62.36 ± 29.88	39.26 ± 33.56	**0** **.** **033**
Duration of hospitalization, days	83.00 ± 29.11	60.02 ± 47.28	**0** **.** **041**
Intubation need beyond 28 days, *n* (%)	33 (32)	15 (5.7%)	**0** **.** **003**
Extubation success following first steroid course, *n* (%)	33 (32.5)	145 (54.7)	**0** **.** **009**
Oxygen need at 36, weeks, *n* (%)			
No	21 (20.4%)	174 (65.6%)	**0** **.** **009**
Oxygen with noninvasive ventilatory support	78 (75.7%)	85 (32.1%)	**0** **.** **012**
Oxygen with invasive ventilatory support	4 (3.9%)	6 (2.3%)	0.487
Moderate or severe BPD	82 (79.6%)	91 (34.4%)	**0** **.** **013**
Mortality after 36, weeks	10 (9.7%)	25 (9.4%)	0.388

Statistically significant p-values are indicated in bold.

BPD, bronchopulmonary dysplasia.

By the 36th gestational week, 4 of the infants in the DART group (3.9%) and 6 of the infants in the enhanced dose group (2.3%) were receiving oxygen through invasive ventilatory support. Oxygen delivery through noninvasive ventilatory support ratio was 75.7% in the DART group and 32.1% in the enhanced low dose group. 65.6% of the infants in the enhanced low dose group was completely free of oxygen support while this ratio was significantly lower (20.4%) in the DART group (*p* = 0.009) (Table 4).

At 36 weeks' corrected gestational age, 3.9% (4/103) of infants in the DART group and 2.3% (6/265) in the enhanced low-dose group remained on invasive ventilatory support (*p* = 0.487). Noninvasive oxygen delivery was required in 75.7% of the DART group vs. 32.1% of the enhanced low-dose group (*p* = 0.012). Notably, 65.6% of infants in the enhanced low-dose group were completely free of oxygen support, significantly higher than the 20.4% observed in the DART group (*p* = 0.009) (Table 4).

In the study cohort, 3 infants in the DART group were discharged home xith oxygen support while number was only one in the enhanced low dose group (*p* = 0.087). 66 infants had died. The most common causes of death were immaturity accompanied by RDS (27.3%), sepsis (22.7%), pulmonary hemorrhage (15.1%), and intractable hypotension with poor perfusion (9.1%). 31 of the infants died before 36th corrected gestational weeks predominantly due to RDS and pulmonary hemorrhage and 35 died after 36th corrected gestational weeks, predominantly due to sepsis and/or intractable hypotension.

## Discussion

BPD remains a significant challenge in neonatology, and despite decades of research, the optimal steroid regimen for its management is still debated. Our study aimed to compare the respiratory outcomes of premature infants treated with an enhanced low-dose systemic dexamethasone regimen (1.35 mg/kg) vs. the conventional DART protocol (0.89 mg/kg). The primary objective of assessing the effectiveness of a higher cumulative steroid dose was achieved with promising results. The findings suggest that increasing the cumulative dexamethasone dose within the low-dose range may lead to improved respiratory outcomes without significantly increasing adverse effects.

One of the key findings of our study was the significant reduction in the duration of both invasive and noninvasive ventilatory support in the enhanced low-dose group compared to the DART group. By the 36th postmenstrual week, a significantly higher proportion of infants in the enhanced low-dose group were completely off oxygen support (65.6% vs. 20.9%, *p* = 0.009). These results align with previous studies suggesting that moderately early initiation and slightly higher cumulative doses of dexamethasone may be more effective in facilitating extubation and reducing the severity of BPD (13, 18, 23).

Although the DART protocol (cumulative dose: 0.89 mg/kg) is the most commonly preferred therapy in NICUs across Turkey, it has already been shown that higher-dose regimens of dexamethasone, administered after the first week of life, are more effective in reducing the risk of BPD without increasing the risk of neurodevelopmental sequelae in ventilated preterm infants (24). Previously, the MINIDEX trial was designed to compare very low-dose dexamethasone (13 × 0.05 mg/kg/dose, cumulative dose: 0.65 mg/kg) with placebo. However, due to poor recruitment and a high discontinuation rate, the trial was discontinued (23). Al-Taweel et al. compared low-dose dexamethasone therapy (DART) with an enhanced low-dose regimen. The extubation success rate was 45.6% in the DART group and 53,3% in the enhanced low-dose group. In our study, the extubation success rate was significantly higher in the enhanced low-dose group (55% vs. 32.4%; *p* = 0.009) with a much shorter duration of invasive ventilatory support compared to the DART group (6.87 ± 13.93 vs. 18.48 ± 14.13 days, *p* = 0.021).This outcome result is also surpassing the results of studies investigating the DART protocol (21). This may be partly attributed to differences in the mean gestational ages of infants between these studies, as well as variations in clinical practices for facilitating extubation.

A meta-analysis of six studies involving 209 participants compared higher (>2.7 mg/kg) and lower (≤2.7 mg/kg) dexamethasone dose regimens. The analysis found no significant effect of dosage on death or neurodevelopmental outcomes, but higher doses were more effective in reducing chronic lung disease. However, the authors concluded that small sample sizes, study heterogeneity, and limited long-term data complicated interpretation (22). In another trial, a long-duration (42-day) high cumulative dose (7.98 mg/kg) of dexamethasone demonstrated better neurodevelopmental outcomes compared to a shorter-duration (9-day) regimen with a lower cumulative dose (2.63 mg/kg). Unfortunately, this trial was also prematurely halted (25).

A major concern regarding systemic corticosteroid use in preterm infants is the risk of adverse effects, including hypertension, hyperglycemia, gastrointestinal complications, and long-term neurodevelopmental impairment (16, 17). In our cohort, only four infants-three in enhanced low dose group, one in the DART group-developed transient hypertension, and one had interventricular septal hypertrophy; all cases resolved spontaneously without intervention. Notably, no instances of hyperglycemia, electrolyte disturbances, or gastrointestinal complications were observed. These findings suggest that increasing the cumulative dexamethasone dose within the low-dose range may not significantly elevate the risk of short-term steroid-related complications. However, the potential for long-term neurodevelopmental sequelae warrants ongoing evaluation.

In contrast, Padam et al. reported steroid-associated complications in 91% of 47 neonates treated with dexamethasone, including increased rates of culture-positive infections (53%), hypoglycemia (23%), hyperglycemia (23%), hypertension requiring treatment (4%), gastrointestinal bleeding (6%), and suspected or confirmed necrotizing enterocolitis (6%) (26). Additional morbidities such as PDA, ROP, hypertrophic cardiomyopathy, periventricular leukomalacia, and death were also documented, though the study did not clarify whether these outcomes were significantly more frequent among steroid-exposed infants. Given the baseline vulnerability of preterm neonates to these conditions, we believe that causality is difficult to establish without controlled comparisons.

Historically, systemic corticosteroids—especially dexamethasone—have been associated with concerns about severe adverse outcomes such as cerebral palsy, neurosensory disabilities, and gastrointestinal perforation (e.g., Yeh et al., 2004; Doyle et al., 2005) (27, 28). However, the most recent Cochrane review (27, 29) concluded that when administered after the first week of life, low-dose corticosteroids do not significantly increase the risk of these outcomes in most trials. Moreover, a recent meta-analysis by Been et al. found that lower cumulative doses of postnatal dexamethasone were associated with reduced risk of bronchopulmonary dysplasia without significant neurodevelopmental harm (30).

Our findings challenge earlier studies linking systemic corticosteroids to high rates of adverse effects and support recent evidence suggesting a more favorable short-term safety profile for modified low-dose regimens. Based on this data, we propose that carefully monitored increases in cumulative corticosteroid exposure—within the established low-dose range—may be considered in select high-risk infants, provided that long-term outcomes continue to be rigorously evaluated.

Despite improved respiratory outcomes, our study did not demonstrate a significant difference in overall prematurity-related complications, including NEC, ROP, or sepsis, between the two groups. This is consistent with existing literature, which suggests that while systemic steroids may improve pulmonary outcomes, their impact on other prematurity-related morbidities remains inconclusive (13, 18).

The mortality rate in our study was comparable to previous reports, with RDS and sepsis being the leading causes of death. While steroid therapy may contribute to improved survival by enhancing pulmonary function, other factors such as infection control, nutritional support, and overall neonatal intensive care practices play crucial roles in determining outcomes.

## Limitations and future directions

While our findings are encouraging, retrospective study design with only historical controls does not allow direct comparison with the DART protocol, limiting definitive conclusions regarding its superiority. Besides, reliance on single-center records may limit generalizability. Additionally, while our findings indicate that a slightly higher dexamethasone dose may be beneficial, the study does not address long-term neurodevelopmental outcomes, which remain a major concern in steroid therapy for preterm infants. In our study, long-term neurodevelopmental outcomes of the patients were not analyzed due to the unavailability of follow-up data. Future prospective studies with larger cohorts and long-term follow-up are needed to confirm our results and determine the safest and most effective corticosteroid regimen for BPD management.

## Conclusion

In conclusion, our study suggests that an enhanced low-dose dexamethasone regimen (1.35 mg/kg) may offer superior respiratory outcomes compared to the conventional DART protocol without a significant increase in short-term adverse effects. However, careful patient selection and close monitoring remain essential to minimize potential risks. These findings contribute to the ongoing debate regarding the optimal corticosteroid regimen for BPD treatment and highlight the need for further larger and multicenter research to refine steroid dosing strategies in preterm infants.

## Data Availability

The original contributions presented in the study are included in the article/Supplementary Material, further inquiries can be directed to the corresponding author.
